# Long‐Term Safety and Efficacy of Pegvaliase in Japanese Adults With Phenylketonuria: Final Results of a Phase III Trial

**DOI:** 10.1002/jmd2.70084

**Published:** 2026-04-08

**Authors:** Yoko Nakajima, Mika Ishige, Tetsuya Ito, Takashi Hamazaki, Mitsuhiro Kuwahara, Lawrence Lee, Haruo Shintaku

**Affiliations:** ^1^ Department of Pediatrics Fujita Health University School of Medicine Toyoake Japan; ^2^ Department of Pediatrics and Child Health Nihon University School of Medicine Tokyo Japan; ^3^ Department of Pediatrics Osaka Metropolitan University Graduate School of Medicine Osaka Japan; ^4^ BioMarin Pharmaceutical Japan K.K. Tokyo Japan; ^5^ BioMarin Pharmaceutical Inc. Novato California USA

**Keywords:** diet, Japan, pegvaliase, PEGylated phenylalanine ammonia lyase, phenylalanine hydroxylase deficiency, phenylketonuria

## Abstract

Phenylketonuria (PKU) is an inborn error of metabolism leading to phenylalanine (Phe) accumulation and consequent neurological, neurocognitive, and psychiatric symptoms. Pegvaliase, a pegylated recombinant phenylalanine ammonia lyase that metabolizes Phe, effectively reduced blood Phe in phase III studies in the United States. This multicenter, open‐label, phase III study (jRCT2080224573) evaluated efficacy and safety of up to 4 years of pegvaliase treatment in 12 adult Japanese participants with PKU (blood Phe > 600 μmol/L). Subcutaneous pegvaliase followed an induction/titration/maintenance dosing regimen up to a maximum of 60 mg/day. After Week 52, diet and pegvaliase dose could be adjusted if blood Phe was ≤ 360 μmol/L. Mean (standard deviation [SD]) treatment duration was 166.4 (66.5) weeks. At Week 192 (*n* = 10), mean (SD) blood Phe was 296.2 (430.7) μmol/L, a 71.2% decrease from baseline, and daily protein intake from intact and medical food was 49.9 (21.4) g (68.0% increase) and 7.6 (16.2) g (64.2% decrease), respectively. All participants had ≥ 1 treatment‐emergent adverse event (TEAE) during induction/titration, most commonly injection site erythema and injection site swelling (83.3% each); nine of 10 had a TEAE during maintenance. Of 395 TEAEs recorded during maintenance, 82 occurred between the 2‐year interim analysis and the 4‐year final analysis. One serious TEAE (allergic arthritis) was considered pegvaliase related. The exposure‐adjusted rate of pegvaliase‐related events was 17.0 per person‐year (41.2 during induction/titration, 8.9 during maintenance). Pegvaliase effectively lowered blood Phe in Japanese participants with PKU, with no new safety issues with long‐term treatment, and many participants were able to liberalize their diet.

## Synopsis

- Pegvaliase lowered blood phenylalanine in Japanese participants with phenylketonuria, providing the opportunity for many participants to liberalize their diet.

## 1 Introduction

Phenylketonuria (PKU) is an inborn error of metabolism in which deficiency of the enzyme phenylalanine hydroxylase (PAH) leads to accumulation of phenylalanine (Phe) in blood and tissues [1]. High Phe levels can lead to a range of neurological, neurocognitive, and psychiatric symptoms [1, 2, 3, 4]. The prevalence of PKU is approximately 1:23 930 live births globally [5]. In Japan, the incidence of PKU is estimated to be 1:70 000 live births [6], and after the introduction of tandem mass spectrometry for screening, the incidence was reported to be approximately 1:46 000, including mild hyperphenylalaninemia [7]. PKU has historically been managed through medical nutrition therapy (MNT), an approach that encompasses three key components: a Phe‐restricted diet, supplementation with Phe‐free protein sources, and the inclusion of low‐protein modified foods. However, adhering to a Phe‐restricted diet is challenging and negatively affects quality of life [1, 8, 9]. Moreover, guideline‐recommended blood Phe levels of 30–360 μmol/L [6, 10, 11, 12, 13] are achieved by only a minority of patients treated with MNT alone [14, 15].

In Japan, patients with PKU who are responsive to the enzyme cofactor tetrahydrobiopterin (BH4) can be treated with sapropterin dihydrochloride, a synthetic form of BH4 [10]. However, between 44% and 80% of patients do not respond to sapropterin [16, 17]. In a long‐term postmarketing surveillance study in Japan, sapropterin was judged by the treating physicians to be effective in reducing blood Phe levels in more than 90% of enrolled patients with BH4‐responsive PKU [18]. However, despite this success, many patients did not achieve recommended blood Phe levels [18].

Pegvaliase (Palynziq, BioMarin Pharmaceutical Inc.), a pegylated recombinant phenylalanine ammonia lyase (PAL) derived from *Anabaena variabilis* that metabolizes Phe to trans‐cinnamic acid and ammonia, has been extensively evaluated in the phase III PRISM studies conducted in the United States [19, 20, 21, 22]. In the PRISM studies, an induction/titration/maintenance (I/T/M) pegvaliase dosing regimen was used to maximize tolerability and guide patients to the randomized target dose [19, 20, 21, 22]. Only after achieving stable dosing in the open‐label extension could adjustments be made to align with individualized blood Phe goals. This I/T/M dosing regimen resulted in statistically significant, clinically meaningful reductions in blood Phe with pegvaliase treatment compared with placebo, with an acceptable safety profile [19]. Importantly, efficacy was maintained over the long term (mean 36.6 months), with 84.7% of blood Phe responders achieving a sustained Phe response at ≤ 360 μmol/L [20]. The outcome of the PRISM clinical development program has resulted in the approval of pegvaliase in multiple geographic regions, including in Japan in March 2023.

In June 2019, a multicenter, open‐label, phase III study (Study 165–305) was initiated to evaluate the efficacy and safety of pegvaliase in Japanese participants aged ≥ 18 years with PKU (blood Phe > 600 μmol/L). The 2‐year (up to Week 144) interim analysis results were previously published [23]. In this article, we report the final results, including changes in blood Phe, dietary changes, pegvaliase dose optimization, and safety, over 4 years (up to Week 208) of pegvaliase treatment.

## 2 Materials and Methods

### 2.1 Study Design

This was an open‐label, multicenter, phase III study of the efficacy and safety of pegvaliase in Japanese adults with PKU conducted between June 13, 2019, and August 23, 2023. Details of the study design were previously described in a 2‐year interim report [23]. In brief, the study consisted of a 4‐week screening and baseline period that preceded a 52‐week I/T/M pegvaliase dosing period (Part 1) and a long‐term extension period lasting up to 168 weeks (Part 2) (Figure S1).

The study was registered at the Japan Registry of Clinical Trials (ID: jRCT2080224573). The study protocol was approved by the Institutional Review Board at each study site. The study was conducted in accordance with relevant national and local regulations, the Japanese Ministerial Ordinance on Good Clinical Practice for Drugs, and ethical principles established by the Declaration of Helsinki. All participants provided written informed consent before any study procedures were undertaken.

### 2.2 Study Population

Japanese adults with PKU aged ≥ 18 to ≤ 70 years with blood Phe levels > 600 μmol/L were eligible for enrollment. Exclusion criteria included the following: any previous pegvaliase treatment; sapropterin treatment within 14 days before starting Part 1; treatment with any other PKU medication (other than MNT) within 2 days before Part 1; pregnancy or breastfeeding, or intention to become pregnant (self or partner); and any disease or condition considered a safety risk.

### 2.3 Pegvaliase Treatment

During the induction phase, pegvaliase was administered subcutaneously at 2.5 mg once a week for 4 weeks. During the titration phase, pegvaliase dose and frequency of administration were increased to 10 mg/day, with the dosing regimen guided by blood Phe levels and safety. During maintenance (up to Week 52 in Part 1), doses could be further increased up to 40 mg/day. During Part 2, if blood Phe was > 360 μmol/L after 16 weeks or more at 40 mg/day (Part 1 and/or Part 2), pegvaliase could be increased up to 60 mg/day.

### 2.4 Other Medications

During Part 1, all participants received an H1‐ or H2‐receptor antagonist, with or without an antipyretic, 2–3 h before each pegvaliase dose; other pre‐medication could be used at the investigator's discretion. All participants could take supplemental tyrosine at the investigator's discretion. In the global PRISM studies, participants were instructed to take 1500 mg/day supplemental tyrosine per protocol; however, as blood tyrosine levels were not related to the level of adherence,^21^ tyrosine supplementation was optional in the current study.

### 2.5 Dietary Restrictions

During Part 1, participants were asked to maintain stable dietary protein from medical food and intact food sources, logged using a 3‐day diet diary. Participants with two consecutive blood Phe values < 30 μmol/L (hypophenylalaninemia; HypoPhe) first adjusted their medical food and intact protein intake, followed by pegvaliase dose adjustment, as advised by the investigator. During Part 2, participants with blood Phe levels ≥ 30 to ≤ 360 μmol/L could decrease medical food intake and increase intact protein intake in consultation with a study dietitian.

### 2.6 Outcome Measures

Efficacy and safety were co‐primary objectives of the study. The primary efficacy endpoint, reported previously [23], was the change in blood Phe levels from baseline to Week 52 (end of Part 1). Participants were instructed to fast at least 2.5 h prior to blood draws. Plasma samples were processed following standardized procedures, including freezing at −70°C within 30 min of sample collection, before measurement of Phe and tyrosine levels at a central laboratory. Exploratory efficacy endpoints included the change in blood Phe levels, the change in dietary protein intake from intact food and medical food sources, and the change in Attention Deficit Hyperactivity Disorder Rating Scale (Investigator‐rated) (ADHD‐RS‐IV) Inattention subscale over time from baseline through the end of Part 2. Safety was evaluated through the incidence of treatment‐emergent adverse events (TEAEs), serious adverse events (SAEs), and adverse events of special interest (AESIs), summarized by Medical Dictionary for Regulatory Activities (Version 24.0) System Organ Class, Preferred Term, relationship to study drug, and severity. AESIs included hypersensitivity, anaphylaxis, and skin reactions lasting ≥ 14 days without improvement. Exposure‐adjusted event rates were calculated by dividing the total number of events by the total treatment exposure (in person‐years) in the period under assessment for the participants in the population being assessed. The exposure time for each participant was derived as the time from the date of first dose to the date of the last dose in the period under assessment. Immunogenicity testing for anti‐PAL immunoglobulin G (IgG), anti‐PAL immunoglobulin M (IgM), anti‐polyethylene glycol (PEG) IgG, anti‐PEG IgM, pegvaliase‐neutralizing antibodies (NAb), and total anti‐pegvaliase antibodies (TAb) was also conducted throughout the study.

### 2.7 Statistical Analysis

As described previously [23], the planned sample size was 10 participants. The Efficacy Population included all participants who received ≥ 1 pegvaliase dose and had ≥ 1 post‐treatment blood Phe measurement. The Efficacy Evaluable Population included all participants who completed 52 weeks of pegvaliase treatment and had a blood Phe measurement at Week 52. The Safety Population included all participants who received ≥ 1 pegvaliase dose. SAS 9.2 or later (SAS Institute Inc., Cary, NC, USA) was used for all statistical analyses.

## 3 Results

### 3.1 Demographic and Baseline Clinical Characteristics

As reported previously, 12 participants were enrolled in the study (Table 1); of these, one participant (Participant 12) withdrew during Part 1, and two participants withdrew during Part 2 (Participants 1 and 9) [23]. Although Participant 9 discontinued pegvaliase treatment, safety follow‐up was continued through the end of study. All participants were included in the Safety Population. Participant 12 was excluded from the Efficacy Evaluable Population because they did not complete Part 1.

**TABLE 1:** Summary of outcomes by participant in order of time to first achievement of blood Phe ≤ 360 μmol/L.

Participant details				Blood Phe (μmol/L)		First achievement of ≤ 360 μmol/L		Pegvaliase administration			Daily protein intake (g)					
ID	Age (years)	Sex	Weight (kg)	Base‐line	Final	Daily dose (mg)	Time to reach (weeks)	Duration (weeks)	At last exposure		Baseline			Last follow‐up^a^		
									Dose (mg)	Frequency	Total	Intact food (%)	Medical food (%)	Total	Intact food (%)	Medical food (g [%])
3	31	F	54	774	4	20	16	212	10	2×/week	68.7	19.7 (28.7)	49.0 (71.3)	74.7	57.7 (77.2)	17.0 (22.8)
6	46	F	54	828	719	20	16	199	10	4×/week	55.2	36.2 (65.6)	19.0 (34.4)	60.2	60.2 (100)	0
2	22	M	56	1113	186	20	36	207	10	2×/week	34.5	17.9 (52.0)	16.6 (48.0)	44.7	44.7 (100)	0
9	32	M	69	1196	683^b^	40	44	63	40	Daily	79.9	79.9 (100)	0	72.2	72.2 (100)	0
11	38	F	49	1112	17	40	44	191	10	Daily	50.7	30.6 (60.3)	20.2 (39.7)	59.6	59.6 (100)	0
10	22	M	64	1142	207	40	48	191	40	Daily	38.6	37.2 (96.5)	1.3 (3.4)	47.7	47.7 (100)	0
1	21	M	59	823	292^b^	40	60	91	40	Daily	63.0	16.1 (25.5)	46.9 (74.5)	57.5	10.6 (18.4)	46.9 (81.6)
7	37	M	78	1149	23	60	68	206	20	Daily	51.0	51.0 (100)	0	37.6	37.6 (100)	0
8	23	F	54	964	< 2	40	72	199	20	Daily	60.4	27.4 (45.3)	33.0 (54.7)	43.9	34.4 (78.4)	9.5 (21.6)
4	31	M	104	1278	227	40	168	207	40	Daily	48.8	47.5 (97.3)	1.3 (2.7)	38.3	38.3 (100)	0
5	20	M	75	904	709	—	—	207	40	Daily	53.4	2.5 (4.6)	50.9 (95.4)	56.4	5.5 (9.8)	50.9 (90.2)
12	30	M	59	1104	1178	—	—	24	20	2×/week	76.1	48.3 (63.4)	27.8 (36.6)	66.6	48.1 (72.2)	18.5 (27.8)

Abbreviations: F, female; M, male; Phe, phenylalanine.

^a^ Final diet status was calculated as the mean of the daily protein intake from intact food and medical food at early termination/study completion.

^b^ Blood Phe at last pegvaliase exposure.

Overall, mean (standard deviation [SD]) baseline age was 29.4 (8.1) years and mean (SD) baseline weight was 64.4 (15.2) kg; four participants were female. At baseline, mean (SD) blood Phe was 1032.3 (166.2) μmol/L, with individual values ranging from 774 to 1278 μmol/L (Table 1). Mean (SD) baseline daily protein intake was 34.5 (20.5) g from intact food and 22.2 (19.6) g from medical food. Ten of the 12 participants used medical food at baseline (Table 1). No participants took tyrosine supplements. Although individual levels of tyrosine slightly lower than the normal range were observed (20–29 μmol/L), none were considered clinically significant, and there were no specific symptoms related to low tyrosine levels. Five participants had low tyrosine at ≥ 2 consecutive visits, but none of these participants experienced alopecia.

### 3.2 Pegvaliase Exposure

Mean (SD) duration of pegvaliase treatment was 166.4 (66.5) weeks (Table 2), compared with 117.2 (38.4) weeks in the interim analysis [23]. Nine participants received pegvaliase for 192–212 weeks (Table 1). One participant (Participant 9) discontinued pegvaliase treatment after Week 64 because of their partner's desire to become pregnant but remained in the study. At the end of the study, the mean (SD) average weekly dose of pegvaliase was 148.5 (79.5) mg/week (Table 2), compared with 151.9 (73.7) mg/week in the interim analysis [23]. The maximum daily dose received was 20 mg in four participants, 40 mg in seven participants, and 60 mg in one participant.

**TABLE 2:** Pegvaliase exposure.

Variable	Safety population (*N* = 12)
Average weekly dose received, mg/week^a^	
Mean (SD)	148.5 (79.5)
Median	172.8
Min, max	34, 235
Total treatment duration, weeks	
Mean (SD)	166.4 (66.5)
Median	199.0
Min, max	24, 212

Abbreviations: max, maximum; min, minimum; SD, standard deviation.

^a^ Average weekly pegvaliase dose received was calculated for each participant by dividing their total cumulative dose by the number of weeks during which they received at least one dose. The mean and SD across all 12 participants were then calculated.

### 3.3 Blood Phe Levels

**FIGURE 1:**
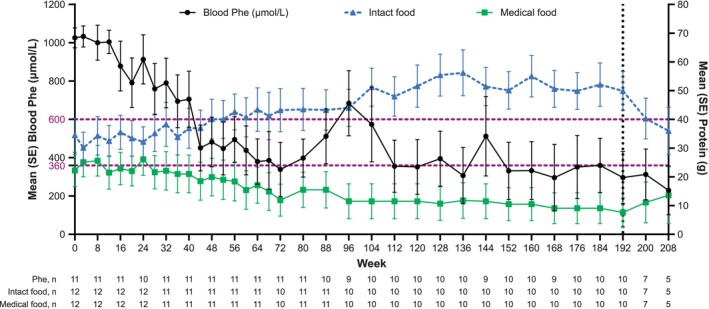
Mean blood Phe and dietary protein from medical food and intact food. The vertical line at Week 192 is to highlight the lower *n*'s at the subsequent time points (Weeks 200 and 208). Phe, phenylalanine; SE, standard error.

**FIGURE 2:**
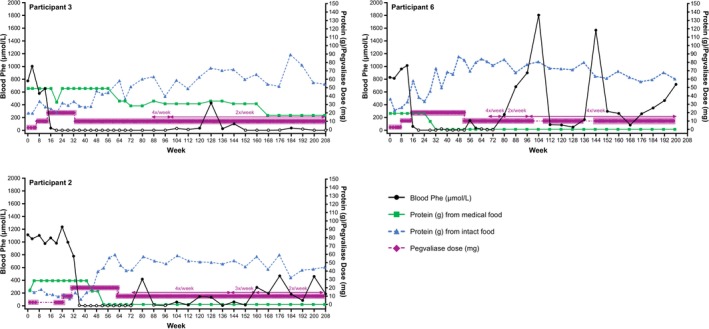
Blood Phe, dietary protein, and pegvaliase dose in individual participants who first achieved blood Phe ≤ 360 μmol/L while on 20 mg/day pegvaliase. Open circles indicate periods of hypophenylalaninemia (two consecutive blood Phe < 30 μmol/L). Prescribed pegvaliase dose is daily except where noted. Phe, phenylalanine.

**FIGURE 3:**
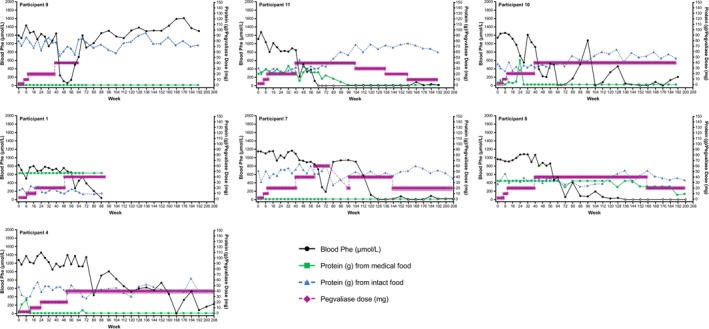
Blood Phe, dietary protein, and pegvaliase dose in individual participants who first achieved blood Phe ≤ 360 μmol/L while on 40 or 60 mg/day pegvaliase. Open circles indicate periods of hypophenylalaninemia (two consecutive blood Phe < 30 μmol/L). Prescribed pegvaliase dose is daily except where noted. Phe, phenylalanine.

**FIGURE 4:**
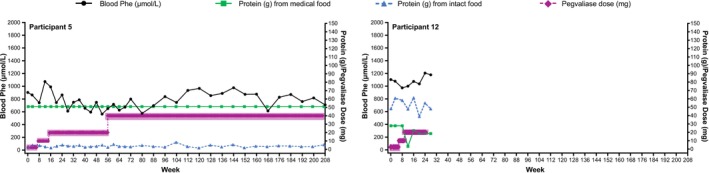
Blood Phe, dietary protein, and pegvaliase dose in individual participants who did not achieve blood Phe ≤ 360 μmol/L. Phe, phenylalanine.

In the Efficacy Evaluable Population, mean (SD) blood Phe at 208 weeks (*n* = 5) was 229.4 (285.6) μmol/L, a decrease of 77.0% (32.0%) from pegvaliase‐naïve baseline (Figure 1). At Week 192 (*n* = 10), mean (SD) blood Phe was 296.2 (430.7) μmol/L, a decrease of 71.2% (39.8%) from baseline. Mean Phe was generally at or below 360 μmol/L from Week 112 onwards, except for a brief increase at Week 144, where it was still < 600 μmol/L. The peaks after Week 72 were due to increases in blood Phe following pegvaliase discontinuation at Week 64 in Participant 9 and pegvaliase interruptions in Participants 6 and 7. Ten participants achieved blood Phe ≤ 360 μmol/L, all of whom also achieved levels of ≤ 120 μmol/L (Table 1; Figure 2; Figure 3). Blood Phe ≤ 360 μmol/L was first achieved at daily pegvaliase doses of 20 mg in three participants, 40 mg in six participants, and 60 mg in one participant. The median time to reach blood Phe ≤ 360 μmol/L was 46 weeks, with the range detailed in Table 1. Pegvaliase dose and/or frequency was reduced in six of these participants, with three participants receiving 10 mg between two and four times a week at last exposure. Two participants were unable to maintain blood Phe ≤ 360 μmol/L at the time of final evaluation due to dosage adjustment (Participant 6) or discontinuation of treatment (Participant 9). Two other participants did not achieve blood Phe ≤ 360 μmol/L (Table 1; Figure 4). One of these participants (Participant 5) was taking 40 mg/day at the end of the study. The other participant (Participant 12) withdrew from the study during Part 1 while taking 20 mg twice a week.

### 3.4 Dietary Protein Intake

Protein intake from intact food sources increased and protein intake from medical food decreased over time (Figure 1). At Week 192, the mean (SD) daily protein intake from intact food and medical food was 49.9 (21.4) g and 7.6 (16.2) g, respectively. This represents a mean (SD) 68.0% (91.5%) increase in protein from intact food and a mean (SD) 64.2% (45.9%) decrease in protein from medical food. At baseline, 10 of the 12 participants consumed medical food; at the last follow‐up, five of these 10 participants were no longer consuming medical food and two had substantially decreased the proportion of protein intake from medical food (Table 1). Of the 10 participants who achieved blood Phe levels ≤ 360 μmol/L, two did not consume medical food at baseline or at last follow‐up, and seven had either stopped or reduced the amount of medical food by the last follow‐up. Per the protocol, diet restrictions were relaxed during Part 1 following HypoPhe in three participants (Participants 2, 3, and 6) (Figure 2). Dietary restrictions were also relaxed after HypoPhe or low blood Phe levels in Participants 8, 10, and 11 during Part 2 (Figure 3).

### 3.5 ADHD‐RS IV Inattention Subscale Scores

As reported previously [23], the mean (SD) ADHD‐RS‐IV Inattention score at baseline (*n* = 11) was 5.0 (4.67), which is below the level for observable symptoms. The mean (SD) change from baseline was −1.0 (1.61) at Week 52 (*n* = 11), −1.1 (2.08) at Week 168 (*n* = 10), and −3.0 (2.58) at Week 208 (*n* = 4).

### 3.6 Safety and Tolerability

All 12 participants in the Safety Population had ≥ 1 TEAE during induction/titration, and nine of 10 (90%) had a TEAE during the maintenance phase (Table 3). Three participants had a TEAE leading to a dose reduction (all during the maintenance phase) and six participants had a TEAE leading to dose interruption (five participants during the induction/titration phase and two participants during maintenance). One participant (Participant 12) decided to withdraw from the study after experiencing adverse events of injection site erythema, injection site swelling, and arthralgia, all of which were considered related to pegvaliase. Overall, the most common TEAEs (by Preferred Term) were injection site erythema and injection site swelling (83.3% of participants for each), arthralgia (75.0%), nasopharyngitis (66.7%), malaise (66.7%), allergic dermatitis (58.3%), injection site pruritus and urticaria (50.0% each), and injection site pain, pyrexia, headache, COVID‐19, complement factor C3 decreased, and complement factor C4 decreased (41.7% each) (Table S1). Except for COVID‐19 and headache, these TEAEs had a higher incidence during induction/titration than during maintenance. Of 395 TEAEs recorded during maintenance, 82 occurred between the interim analysis and the final analysis. Similarly, 28 of 237 treatment‐related TEAEs recorded during maintenance occurred between the interim analysis and the final analysis.

**TABLE 3:** TEAEs by treatment phase.^a^

Variable	Induction/Titration^b^ (*N* = 12)	Maintenance (*N* = 10)	Overall (*N* = 12)
Participants with any TEAE, *n* (%)	12 (100.0)	9 (90.0)	12 (100.0)
TEAEs leading to dose reduction	0	3 (30.0)	3 (25.0)
TEAEs leading to dose interruption	5 (41.7)	2 (20.0)	6 (50.0)
TEAEs leading to study drug discontinuation	0	0	0
TEAEs leading to study discontinuation	0	0	0
Participants with any SAE, *n* (%)	1 (8.3)	3 (30.0)	4 (33.3)
SAEs leading to dose reduction	0	0	0
SAEs leading to dose interruption	0	2 (20.0)	2 (16.7)
SAEs leading to study drug discontinuation	0	0	0
SAEs leading to study discontinuation	0	0	0
Participants with any treatment‐related TEAE, *n* (%)^c^	12 (100.0)	6 (60.0)	12 (100.0)
Treatment‐related SAEs	0	1 (10.0)	1 (8.3)
Participants with any TEAE of CTCAE Grade ≥ 3, *n* (%)	0	3 (30.0)	3 (25.0)
Participants who died, *n* (%)	0	0	0
Exposure‐adjusted TEAEs			
Total treatment exposure, person‐years	11.0	26.7	40.8
TEAEs, *n* (rate per person‐year)^d^	506 (45.8)	395 (14.8)	901 (22.1)
TEAEs leading to dose reduction	0	4 (0.1)	4 (0.1)
TEAEs leading to dose interruption	27 (2.4)	3 (0.1)	30 (0.7)
TEAEs leading to study drug discontinuation	0	0	0
TEAEs leading to study discontinuation	0	0	0
SAEs, *n* (rate per person‐year)^d^	1 (0.1)	5 (0.2)	6 (0.1)
SAEs leading to dose reduction	0	0	0
SAEs leading to dose interruption	0	2 (0.1)	2 (< 0.1)
SAEs leading to study drug discontinuation	0	0	0
SAEs leading to study discontinuation	0	0	0
Treatment‐related TEAEs, *n* (rate per person‐year)^c^, ^d^	455 (41.2)	237 (8.9)	692 (17.0)
Treatment‐related SAEs^c^	0	1 (< 0.1)	1 (< 0.1)
TEAEs of CTCAE Grade ≥ 3, *n* (rate per person‐year)^d^	0	5 (0.2)	5 (0.1)
Deaths, *n*	0	0	0
AESI, *n* (rate per person‐year)^d^			
Anaphylaxis per NIAID/FAAN	0	1 (< 0.1)	1 (< 0.1)
Anaphylaxis per DPARP	0	1 (< 0.1)	1 (< 0.1)
Skin reactions	9 (0.8)	14 (0.5)	23 (0.6)
Hypersensitivity TEAE	66 (6.0)	77 (2.9)	143 (3.5)

Abbreviations: AESI, adverse event of special interest; CTCAE, Common Terminology Criteria for Adverse Events; DPARP, Division of Pulmonary, Allergy, and Rheumatology Products; MedDRA, Medical Dictionary for Regulatory Activities; NIAID/FAAN, National Institute of Allergy and Infectious Diseases/Food Allergy and Anaphylaxis Network; SAE, serious adverse event; TEAE, treatment‐emergent adverse event.

^a^ TEAEs with onset or worsening after the initiation of study drug and up to 30 days after the last dose of study drug were included. TEAEs were coded using MedDRA Version 24.0 and graded for severity using National Cancer Institute CTCAE Version 5.0. Maintenance phase was reached when a participant achieved Phe ≤ 600 μmol/L for ≥ 26 days with a stable dose (≥ 80% same dose) within the period. A period was defined by Phe assessment dates. Induction/Titration occurred at the first dose and ended 1 day before the start of the Maintenance phase.

^b^ Previously reported in Ishige et al. [23].

^c^ Relationship to study drug was assessed by the investigator.

^d^ Event rate = number of events/person‐year.

Overall, four participants reported six treatment‐emergent SAEs: two Grade 2 SAEs of nasopharyngitis and uterine polyp, and four Grade 3 SAEs of allergic arthritis, anaphylactic reaction, diverticulitis intestinal haemorrhagic, and fall. Compared with the interim analysis [23], only two additional SAEs were reported, both in Participant 3: a Grade 2 uterine polyp and a Grade 3 anaphylactic reaction that was assessed by the investigator as not related to pegvaliase; this was considered due to food allergy to peach (prick test positive). The SAE of allergic arthritis was assessed as related to treatment with 60 mg/day pegvaliase and resulted in interruption of pegvaliase treatment (Participant 7); pegvaliase was resumed at 20 mg/day after the SAE improved and was later increased to 40 mg/day with no recurrence of allergic arthritis. The fall was also not related to pegvaliase but resulted in dose interruption for surgery of the fracture (Participant 6).

The total pegvaliase exposure across the study was 40.8 person‐years (Table 3). Exposure was greater during the maintenance phase compared with the induction/titration phase (26.7 vs. 11.0 person‐years). When adjusted for pegvaliase exposure, the rate of TEAEs per person‐year was 22.1 overall (*n* = 901 events), 45.8 (*n* = 506 events) for the induction/titration phase, and 14.8 (*n* = 395 events) for the maintenance phase; the overall and maintenance phase rates decreased since the interim analysis (28.8 and 20.2 per person‐year, respectively) [23]. The rate of SAE per person‐year was low, with an overall rate of 0.1 (*n* = 6 events), showing similar rates for both the induction/titration and maintenance phases. The rate of treatment‐related TEAEs per person‐year was 17.0 overall (*n* = 692 events), 41.2 (*n* = 455 events) for the induction/titration phase, and 8.9 (*n* = 237 events) for the maintenance phase; again, the rates for the overall study and for the maintenance phase decreased since the interim analysis (23.5 and 13.5 per person‐year, respectively) [23]. TEAEs of Common Terminology Criteria for Adverse Events Grade ≥ 3 were only observed during the maintenance phase, with an exposure‐adjusted rate of 0.2 per person‐year (*n* = 5 events). Among AESIs, the rate of hypersensitivity TEAEs per person‐year was 3.5 overall (*n* = 143 events), 6.0 (*n* = 66 events) for the induction/titration phase, and 2.9 (*n* = 77 events) for the maintenance phase. The rate of skin reactions per person‐year was 0.6 overall (*n* = 23 events), 0.8 (*n* = 9 events) for the induction/titration phase, and 0.5 (*n* = 14 events) for the maintenance phase. As noted above, there was one event of anaphylaxis, which occurred during the later part of the maintenance phase, with an overall exposure‐adjusted rate of < 0.1 per person‐year.

During Part 1 (prior to Week 52), three participants (Participants 2, 3, and 6) experienced HypoPhe, and protein intake from intact food was increased; however, blood Phe remained < 30 μmol/L (Figure 2). Of these, two participants (Participants 2 and 6) had a reduction in pegvaliase dose, which resolved the event. The third participant (Participant 3) also had a dose reduction, but HypoPhe persisted through most of the maintenance phase and at study completion. Throughout the entire study, seven of 11 participants experienced HypoPhe. One participant (Participant 2) experienced two events of Grade 1 alopecia related to HypoPhe. The first alopecia event occurred during Week 48 and was reported as resolved during Week 87, and the second event occurred during Week 107 and resolved during Week 140. Although pegvaliase dosing was not changed in direct response to either alopecia event, the dose was reduced from 20 mg/day to 10 mg/day at Week 62, and the frequency of dosing reduced from four times a week to three times a week at Week 142. Diet was liberalized around the time of the first alopecia event.

As previously described [23], all participants developed antibody responses to pegvaliase, most of which peaked approximately 12–24 weeks after pegvaliase initiation (Figure S2).

## 4 Discussion

The final results of this open‐label phase III study demonstrate that pegvaliase substantially reduces blood Phe levels over the long term (up to 208 weeks) in Japanese participants with PKU. Improved blood Phe control allowed most participants to reduce or eliminate their intake of protein from medical food, accompanied by a decrease in pegvaliase dose in six of the 10 (60%) participants who achieved blood Phe ≤ 360 μmol/L. This finding aligns with a previous US real‐world cohort study, which reported that 12 months after reaching the maximum dose, 35% of patients had reduced their dose, with an average decrease of 46.8% from the maximum dose [24]. In the present study, long‐term pegvaliase treatment was generally well tolerated with no new safety issues identified. These results support the use of pegvaliase in Japanese patients with PKU.

The reduction in blood Phe observed at Week 52, the primary endpoint of the study [23], was generally maintained through Week 208, with mean levels near or below 360 μmol/L from Week 112 onwards. Transient increases in mean blood Phe seen at Weeks 88–104 were the result of dose interruptions in several participants. Overall, the efficacy results were consistent with those of the phase III PRISM trials [20, 22]. Examination of individual participant blood Phe profiles revealed that while some participants responded rapidly to pegvaliase, others required prolonged treatment and/or higher doses before blood Phe decreased. In the PRISM trials, Kaplan–Meier analysis revealed that the probability of achieving a clinically significant blood Phe reduction to ≤ 600, ≤ 360, or ≤ 120 μmol/L at least once by 6 months was 42.8%, 30.1%, and 22.4%, respectively, which increased to 93.0%, 90.8%, and 86.2% at 48 months [20]. This likely reflects variability in the immune response and the time required to develop immune tolerance to pegvaliase, resulting in lower drug clearance and greater exposure, which in turn increases efficacy at any given dose [25]. By comparison, the proportion of participants in the present study who achieved Phe blood levels ≤ 600, ≤ 360, and ≤ 120 μmol/L was 25.0%, 16.7%, and 16.7%, respectively, by Week 28 (~6 months), increasing to 91.7%, 83.3%, and 83.3% by Week 208 (~48 months) (data not shown). Notably, when pegvaliase was interrupted or stopped in several participants, blood Phe levels increased back to baseline levels or above. However, once pegvaliase dosing resumed, blood Phe levels decreased again, indicating that brief interruptions in treatment are possible when required without loss of responsiveness. Achievement of continued blood Phe control and immune tolerization allowed for pegvaliase dose to be decreased in most participants.

Most of the participants in this study who achieved blood Phe ≤ 360 μmol/L did so on daily doses of 20 or 40 mg, often with subsequent down‐dosing to 10 or 20 mg a day and/or reduction of dosing frequency. Interestingly, these doses tended to be lower than the corresponding doses seen in the PRISM studies [20, 22], which may be related to the lower baseline body mass index of Japanese participants compared with their US counterparts (mean of 23.5 vs. 28.4 kg/m^2^). In this study, the effective doses and the final doses were generally lower in participants who weighed < 60 kg than in those weighing > 60 kg. However, it is important to note that evidence from phase II studies supported a change from weight‐based dosing to the I/T/M dosing regimen, and results from the current study are limited by the small number of participants.

The continued reduction in blood Phe allowed for reduction in the use of medical food and diet liberalization in many participants. In most cases, diet liberalization became possible after blood Phe levels were in the HypoPhe range, as specified in the study protocol. This is consistent with analyses of the relationship between blood Phe and diet in the PRISM trials [21]. Additional modeling of data from the PRISM trials suggests that diet liberalization is most likely to succeed after sustained low blood Phe levels are observed, and that stepwise down‐dosing of pegvaliase should only be attempted after diet is liberalized [25]. The individual participant results from this study support this proposed recommendation for long‐term management of patients with PKU. The continuation of medical food at the last follow‐up in some participants may reflect personal and/or clinician preferences for the pace of dietary change, e.g., to fine‐tune daily protein intake or in preparation for a planned pregnancy, as well as day‐to‐day fluctuations in dietary intake. Moreover, although some participants experienced transient low levels of tyrosine in parallel with HypoPhe, no participant took tyrosine supplements. This reflects the clinical priority of lowering blood Phe levels to the point where the intake of intact food can be increased, which then provides a natural source of tyrosine.

As demonstrated in the PRISM studies [26], formation of circulating immune complexes (CICs) of pegvaliase and antidrug antibodies is highest in the early treatment phase (induction/titration), which is reflected in the peak of anti‐PEG and anti‐PAL IgG and IgM titers and in transient reductions in measured free C3/C4 due to complement activation and binding within CICs. As the immune response evolves, anti‐PEG IgG and IgM levels return to baseline. Anti‐PAL IgG and IgM elevations are maintained throughout treatment but are less efficient at activating complement or forming CIC due to the masking of PAL with PEG on the drug's surface [26], leading to normalized free C3/C4 and reduced immunogenicity (i.e., TEAEs) during the maintenance phase.

In the PRISM studies, reduction of blood Phe levels with pegvaliase treatment was associated with improved attention, as measured with the ADHD‐RS‐IV Inattention subscale, particularly in participants with the highest baseline inattention scores and those with the greatest blood Phe reductions [27]. In the current study, the mean baseline ADHD‐RS‐IV Inattention score of 5.0 was well below the level for observable symptoms (scores ≥ 10) [27]. Thus, although some improvement in inattention scores was observed, the low level of impairment at baseline suggests that these changes were unlikely to be clinically relevant in this small population of Japanese patients with PKU.

Long‐term pegvaliase treatment was well tolerated in these Japanese participants.

As noted in the interim report [23], all participants experienced ≥ 1 TEAE, and the incidence of TEAEs was higher during the induction/titration phase than during the maintenance phase. This pattern may be attributed to antibody responses to pegvaliase, which peaked 3–6 months after treatment initiation. TAb, PAL IgG, PAL IgM, and NAb responses remained stable thereafter, while PEG IgG and PEG IgM responses declined toward baseline. Blood Phe continued to decrease despite the presence of sustained levels of NAb, suggesting that these antibodies were only partially neutralizing enzymatic activity in participants. With immune maturation and continued exposure, the incidence of TEAEs declined during the maintenance phase, even as total pegvaliase exposure increased from 15.5 person‐years in the 2‐year interim analysis [23] to 26.7 person‐years in the present study. Compared with the interim analysis [23], there were only two additional SAEs reported, both in the same participant: a Grade 2 uterine polyp, and a Grade 3 anaphylactic reaction that was deemed unrelated to pegvaliase. Overall, these results indicate that long‐term treatment with pegvaliase is not associated with increased incidence of TEAEs, supporting its favorable risk–benefit profile for prolonged use.

This is the first study of the efficacy and safety of long‐term pegvaliase treatment in Japanese participants with PKU. Although only 12 participants were enrolled, the results reflect the breadth of individual experiences observed clinically. Moreover, most participants remained in the study for the full 208 weeks, allowing extended, long‐term assessment. Only one participant withdrew, suggesting that most participants preferred to continue pegvaliase treatment. Despite the study's relatively small sample size, its strength lies in the detailed examination of blood Phe levels, dietary composition, and pegvaliase dose over time, highlighting the need for individualized treatment based on where participants start and their unique trajectory toward achieving optimal outcomes, including diet liberalization and dose optimization.

In conclusion, the results of this study demonstrate that pegvaliase was effective in lowering blood Phe in Japanese participants with PKU, with no new safety issues with long‐term treatment. Moreover, achieving continued blood Phe control allowed many participants to reduce or eliminate their use of medical food, increase protein intake from intact food, and progressively reduce their pegvaliase dose over time.

## Author Contributions

All authors participated in the interpretation of study results, and in the drafting, critical revision, and approval of the final version of the manuscript. M.K. was involved in the study design and was the medical monitor. Y.N., M.I., T.I., T.H., and H.S. were investigators in the study and were involved in data collection. Lawrence Lee conducted the statistical analysis. Y.N. serves as guarantor for the article.

## Funding

This study was funded by BioMarin Pharmaceutical Inc. Medical writing assistance was provided by Rebecca Lew, PhD, CMPP, and Jason Vuong, BPharm, CMPP, of ProScribe KK, part of Envision Ignite, an Envision Medical Communications agency, a part of Envision Pharma Group, and was funded by BioMarin. ProScribe's services complied with international guidelines for Good Publication Practice (GPP). BioMarin was involved in the study design, data collection, data analysis, and preparation of the manuscript.

## Ethics Statement

The study was registered at the Japan Registry of Clinical Trials (ID: jRCT2080224573). The study protocol was approved by the Institutional Review Board at each study site. The study was conducted in accordance with relevant national and local regulations, the Japanese Ministerial Ordinance on Good Clinical Practice for Drugs, and ethical principles established by the Declaration of Helsinki.

## Consent

All participants provided written informed consent before any study procedures were undertaken.

## Conflicts of Interest

Yoko Nakajima, Mika Ishige, Tetsuya Ito, Takashi Hamazaki, and Haruo Shintaku have participated as clinical trial investigators for BioMarin and received speaker fees and travel support from BioMarin. Mika Ishige also reports receiving speaker fees, honoraria, and/or travel support from Daiichi Sankyo Co. Ltd., Otsuka Pharmaceutical Co. Ltd., and PTC Therapeutics Inc. Takashi Hamazaki also reports participating in advisory boards for BioMarin and PTC Therapeutics Inc., receiving clinical trial support and travel support from BioMarin, PTC Therapeutics Inc., and Otsuka Pharmaceutical Co. Ltd., and receiving honoraria for lectures from Daiichi Sankyo Co. Ltd. Lawrence Lee is an employee and stockholder of BioMarin. Mitsuhiro Kuwahara was an employee and stockholder of BioMarin at the time the study was conducted.

## Supporting information


**TABLE S1:** Incidence of TEAEs by System Organ Class, Preferred Term, and treatment phase^a^.
**FIGURE S1:** Design of Study 165–305 (adapted from M. Ishige, et al., Mol. Genet. Metab. 140 [2023] 107697). ^a^Intensive PK sampling taken at pre‐dose, 2, 4, 8, 12, and 24 h post dose. The 24‐h sample was taken prior to the next daily dose. Intensive PK samples were taken in all participants at Week 52 of Part 1. In Part 2, intensive PK samples were taken only in participants receiving 60 mg/day after 8 weeks on 60 mg/day. Phe, phenylalanine; PK, pharmacokinetics.
**FIGURE S2:** Antibody profiles, showing mean levels of total, neutralizing, anti‐PAL, and anti‐PEG antibodies. ^a^ *n* = 10 at Week 56 for NAb, PAL IgM, and PEG IgM; *n* = 11 at Week 56 for PAL IgG, PEG IgG, and TAb. IgG, immunoglobulin G; IgM, immunoglobulin M; NAb, neutralizing antibodies; PAL, phenylalanine ammonia lyase; PEG, polyethylene glycol; TAb, total antibodies.

## Data Availability

The de‐identified individual participant data that underlie the results reported in this article (including text, tables, figures, and appendices) will be made available, together with the research protocol and data dictionaries, for non‐commercial, academic purposes. Additional supporting documents may be available upon request. Investigators will be able to request access to these data and supporting documents via a data sharing portal (www.BioMarin.com/patients/publication‐data‐request) beginning 6 months after and ending 2 years after publication. Data associated with any ongoing development program will be made available within 6 months after approval of relevant product. Requests must include a research proposal clarifying how the data will be used, including proposed analysis methodology. Research proposals will be evaluated relative to publicly available criteria available at www.BioMarin.com/patients/publication‐data‐request to determine if access will be given, contingent upon execution of a data access agreement with BioMarin Pharmaceutical Inc.
