# Supplementary material for: Long‐Term Safety and Efficacy of Pegvaliase in Japanese Adults With Phenylketonuria: Final Results of a Phase III Trial
Source: JIMD Rep. 2026 Apr 8;67(3):e70084. doi: 10.1002/jmd2.70084 (PMC13061746; doi:10.1002/jmd2.70084)

SUPPLEMENTARY MATERIAL

Title

Long-term Safety and Efficacy of Pegvaliase in Japanese Adults With Phenylketonuria: Final Results of a Phase III Trial

Yoko Nakajima^1^, Mika Ishige^2^, Tetsuya Ito^1^, Takashi Hamazaki^3^, Mitsuhiro Kuwahara^4^, Lawrence Lee^5^, Haruo Shintaku^3^

AUTHORS’ AFFILIATIONS

^1^Department of Pediatrics, Fujita Health University School of Medicine, Toyoake, Japan

^2^Department of Pediatrics and Child Health, Nihon University School of Medicine, Tokyo, Japan

^3^Department of Pediatrics, Osaka Metropolitan University Graduate School of Medicine, Osaka, Japan

^4^BioMarin Pharmaceutical Japan K.K., Tokyo, Japan

^5^BioMarin Pharmaceutical Inc., Novato, CA, USA

CORRESPONDING AUTHOR

Yoko Nakajima, MD, PhD

Department of Pediatrics

Fujita Health University School of Medicine

Toyoake, Japan

Email: yonaka@fujita-hu.ac.jp

LIST OF TABLES AND FIGURES

**Table S1** | Incidence of TEAEs by System Organ Class, Preferred Term, and treatment phase.

**Figure S1** | Design of Study 165-305.

**Figure S2** | Antibody profiles, showing mean levels of total, neutralizing, anti-PAL, and anti-PEG antibodies.

**Table S1** | Incidence of TEAEs by System Organ Class, Preferred Term, and treatment phase^a^.

| **Variable** | **Induction/Titration^b^**  **(N=12)** | **Maintenance**  **(N=10)** | **Overall (N=12)** |
| --- | --- | --- | --- |
| **Participants with any TEAE, n (%)** | **12 (100.0)** | **9 (90.0)** | **12 (100.0)** |
| **Skin and subcutaneous tissue disorders** | **11 (91.7)** | **7 (70.0)** | **12 (100.0)** |
| Allergic dermatitis | 6 (50.0) | 3 (30.0) | 7 (58.3) |
| Urticaria | 6 (50.0) | 2 (20.0) | 6 (50.0) |
| Allergic pruritus | 3 (25.0) | 2 (20.0) | 4 (33.3) |
| Erythema | 3 (25.0) | 1 (10.0) | 3 (25.0) |
| Papule | 2 (16.7) | 0 | 2 (16.7) |
| **Gastrointestinal disorders** | **9 (75.0)** | **7 (70.0)** | **11 (91.7)** |
| Nausea | 3 (25.0) | 1 (10.0) | 4 (33.3) |
| Abdominal distension | 1 (8.3) | 1 (10.0) | 2 (16.7) |
| Abdominal pain | 1 (8.3) | 1 (10.0) | 2 (16.7) |
| Diarrhoea | 1 (8.3) | 1 (10.0) | 2 (16.7) |
| Stomatitis | 1 (8.3) | 1 (10.0) | 2 (16.7) |
| Toothache | 1 (8.3) | 2 (20.0) | 2 (16.7) |
| **General disorders and administration site conditions** | **11 (91.7)** | **7 (70.0)** | **11 (91.7)** |
| Injection site erythema | 10 (83.3) | 4 (40.0) | 10 (83.3) |
| Injection site swelling | 10 (83.3) | 1 (10.0) | 10 (83.3) |
| Malaise | 7 (58.3) | 2 (20.0) | 8 (66.7) |
| Injection site pruritus | 6 (50.0) | 3 (30.0) | 6 (50.0) |
| Injection site pain | 5 (41.7) | 0 | 5 (41.7) |
| Pyrexia | 5 (41.7) | 2 (20.0) | 5 (41.7) |
| Chest discomfort | 2 (16.7) | 1 (10.0) | 2 (16.7) |
| Injection site haemorrhage | 1 (8.3) | 2 (20.0) | 2 (16.7) |
| Injection site papule | 2 (16.7) | 0 | 2 (16.7) |
| Injection site rash | 2 (16.7) | 1 (10.0) | 2 (16.7) |
| Vaccination site joint pain | 0 | 2 (20.0) | 2 (16.7) |
| **Infections and infestations** | **8 (66.7)** | **7 (70.0)** | **10 (83.3)** |
| Nasopharyngitis | 7 (58.3) | 4 (40.0) | 8 (66.7) |
| COVID-19 | 0 | 5 (50.0) | 5 (41.7) |
| Gastroenteritis | 0 | 3 (30.0) | 3 (25.0) |
| Pharyngitis | 1 (8.3) | 1 (10.0) | 2 (16.7) |
| **Musculoskeletal and connective tissue disorders** | **9 (75.0)** | **5 (50.0)** | **10 (83.3)** |
| Arthralgia | 9 (75.0) | 4 (40.0) | 9 (75.0) |
| Back pain | 2 (16.7) | 2 (20.0) | 3 (25.0) |
| Myalgia | 2 (16.7) | 1 (10.0) | 3 (25.0) |
| Pain in extremity | 1 (8.3) | 2 (20.0) | 3 (25.0) |
| Allergic arthritis | 1 (8.3) | 1 (10.0) | 2 (16.7) |
| **Investigations** | **5 (41.7)** | **4 (40.0)** | **8 (66.7)** |
| Complement factor C3 decreased | 5 (41.7) | 1 (10.0) | 5 (41.7) |
| Complement factor C4 decreased | 5 (41.7) | 0 | 5 (41.7) |
| Amino acid level decreased | 0 | 2 (20.0) | 2 (16.7) |
| **Immune system disorders** | **3 (25.0)** | **6 (60.0)** | **7 (58.3)** |
| Allergy to vaccine | 0 | 3 (30.0) | 3 (25.0) |
| Serum sickness-like reaction | 3 (25.0) | 1 (10.0) | 3 (25.0) |
| Hypersensitivity | 2 (16.7) | 2 (20.0) | 2 (16.7) |
| **Nervous system disorders** | **5 (41.7)** | **6 (60.0)** | **6 (50.0)** |
| Headache | 4 (33.3) | 4 (40.0) | 5 (41.7) |
| Dizziness | 1 (8.3) | 2 (20.0) | 2 (16.7) |
| Sciatica | 0 | 2 (20.0) | 2 (16.7) |
| **Respiratory, thoracic and mediastinal disorders** | **4 (33.3)** | **3 (30.0)** | **6 (50.0)** |
| Cough | 1 (8.3) | 2 (20.0) | 2 (16.7) |
| **Injury, poisoning and procedural complications** | **1 (8.3)** | **5 (50.0)** | **5 (41.7)** |
| Ligament sprain | 0 | 2 (20.0) | 2 (16.7) |
| **Eye disorders** | **1 (8.3)** | **3 (30.0)** | **4 (33.3)** |
| Asthenopia | 0 | 2 (20.0) | 2 (16.7) |
| **Reproductive system and breast disorders** | **0** | **3 (30.0)** | **3 (25.0)** |
| **Blood and lymphatic system disorders** | **0** | **2 (20.0)** | **2 (16.7)** |
| **Hepatobiliary disorders** | **1 (8.3)** | **1 (10.0)** | **2 (16.7)** |
| Hepatic steatosis | 1 (8.3) | 1 (10.0) | 2 (16.7) |
| **Vascular disorders** | **1 (8.3)** | **1 (10.0)** | **2 (16.7)** |
| **Cardiac disorders** | **1 (8.3)** | **0** | **1 (8.3)** |
| **Ear and labyrinth disorders** | **0** | **1 (10.0)** | **1 (8.3)** |
| **Endocrine disorders** | **0** | **1 (10.0)** | **1 (8.3)** |
| **Neoplasms benign, malignant and unspecified** | **0** | **1 (10.0)** | **1 (8.3)** |
| **Psychiatric disorders** | **0** | **1 (10.0)** | **1 (8.3)** |
| **Renal and urinary disorders** | **0** | **1 (10.0)** | **1 (8.3)** |

Abbreviations: MedDRA, Medical Dictionary for Regulatory Activities; Phe, phenylalanine; TEAE, treatment-emergent adverse event.

^a^TEAEs with onset or worsening after the initiation of study drug and up to 30 days after the last dose of study drug were included. TEAEs were coded using MedDRA Version 24.0. Participants with ≥1 TEAE of the same System Organ Class/Preferred Term were counted only once for that System Organ Class/Preferred Term. Preferred Terms are shown only for TEAEs occurring in ≥10% of participants overall. Maintenance phase was reached when a participant achieved Phe ≤600 µmol/L for ≥26 days with a stable dose (≥80% same dose) within the period. A period was defined by Phe assessment dates. Induction/Titration occurred at the first dose and ended 1 day before the start of the Maintenance phase.

^b^Previously reported in Ishige M, et al, 2023 [23].

**Figure S1** | Design of Study 165-305 (adapted from M. Ishige, et al, Mol. Genet. Metab. 140 [2023] 107697). ^a^Intensive PK sampling taken at pre-dose, 2, 4, 8, 12, and 24 hours post dose. The 24-hour sample was taken prior to the next daily dose. Intensive PK samples were taken in all participants at Week 52 of Part 1. In Part 2, intensive PK samples were taken only in participants receiving 60 mg/day after 8 weeks on 60 mg/day. Phe, phenylalanine; PK, pharmacokinetics.


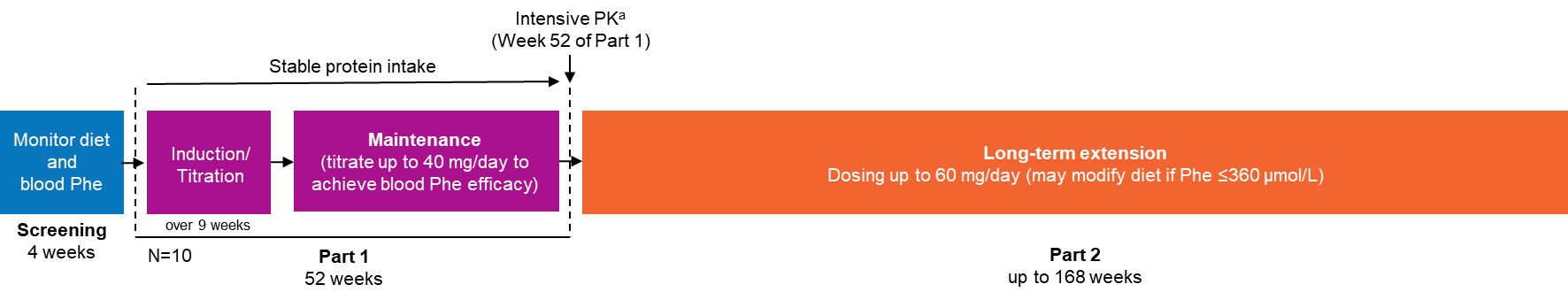


**Figure S2** | Antibody profiles, showing mean levels of total, neutralizing, anti-PAL, and anti-PEG antibodies. ^a^n=10 at Week 56 for NAb, PAL IgM, and PEG IgM; n=11 at Week 56 for PAL IgG, PEG IgG, and TAb. IgG, immunoglobulin G; IgM, immunoglobulin M; NAb, neutralizing antibodies; PAL, phenylalanine ammonia lyase; PEG, polyethylene glycol; TAb, total antibodies.


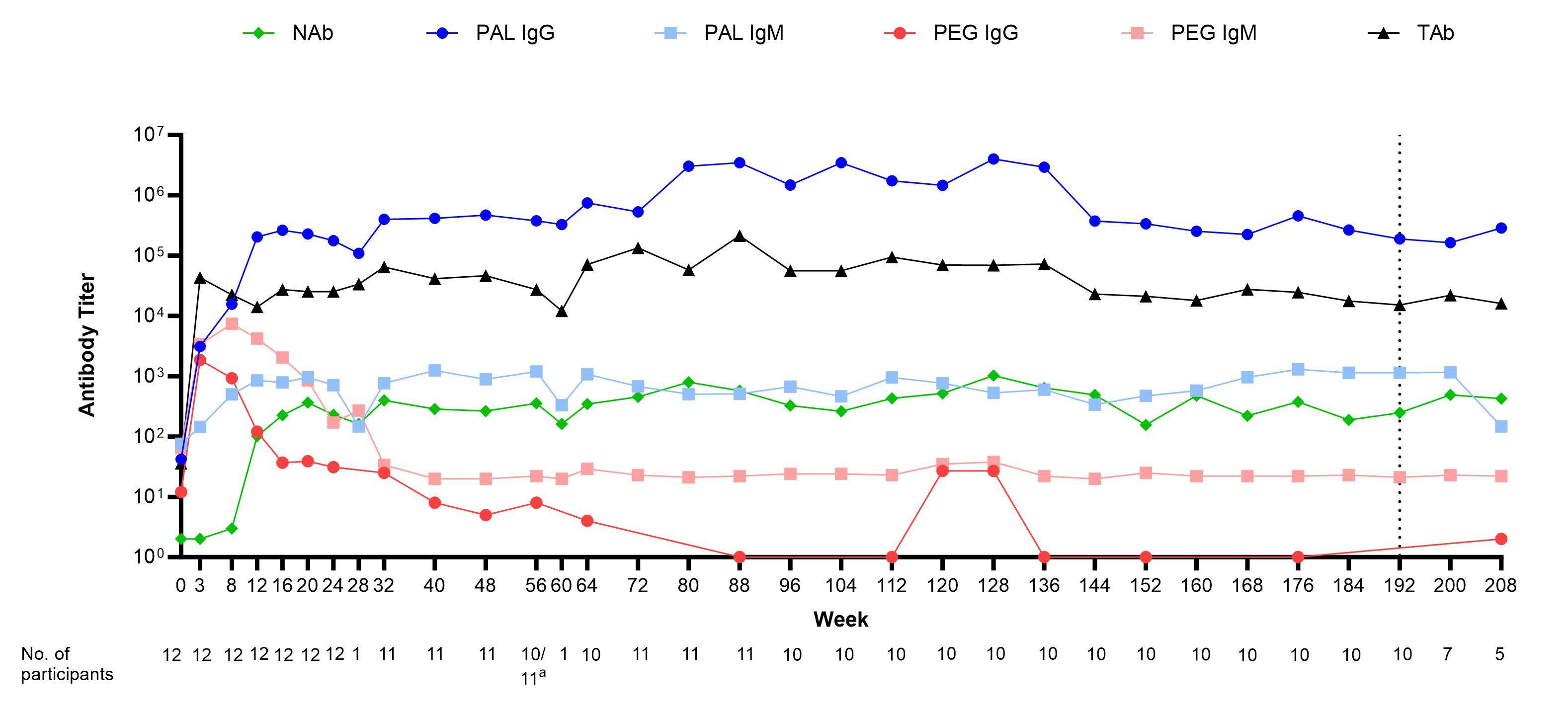

Supplement: Supplementary file 1 — TABLE S1: Incidence of TEAEs by System Organ Class, Preferred Term, and treatment phasea. FIGURE S1: Design of Study 165–305 (adapted from M. Ishige, et al., Mol. Genet. Metab. 140 [2023] 107697). aIntensive PK sampling taken at pre‐dose, 2, 4, 8, 12, and 24 h post dose. The 24‐h sample was taken prior to the next daily dose. Intensive PK samples were taken in all participants at Week 52 of Part 1. In Part 2, intensive PK samples were taken only in participants receiving 60 mg/day after 8 weeks on 60 mg/day. Phe, phenylalanine; PK, pharmacokinetics. FIGURE S2: Antibody profiles, showing mean levels of total, neutralizing, anti‐PAL, and anti‐PEG antibodies. a n = 10 at Week 56 for NAb, PAL IgM, and PEG IgM; n = 11 at Week 56 for PAL IgG, PEG IgG, and TAb. IgG, immunoglobulin G; IgM, immunoglobulin M; NAb, neutralizing antibodies; PAL, phenylalanine ammonia lyase; PEG, polyethylene glycol; TAb, total antibodies. [file JMD2-67-e70084-s001.docx]
